# The construct validity and internal consistency of QuickDASH in pediatric patients with upper extremity fractures

**DOI:** 10.2340/17453674.2024.40181

**Published:** 2024-04-30

**Authors:** Niko KÄMPPÄ, Sina HULKKONEN, Petra GRAHN, Topi LAAKSONEN, Jussi REPO

**Affiliations:** 1 Department of Hand Surgery, Helsinki University Hospital and University of Helsinki; 2 Department of Pediatric Orthopedics and Traumatology, New Children’s Hospital, HUS Helsinki University Hospital, University of Helsinki; 3 Unit of Musculoskeletal Surgery, Department of Orthopedics and Traumatology, Tampere University Hospital and Tampere University, Finland

## Abstract

**Background and purpose:**

Investigation of treatment options in the pediatric population necessitates the use of valid patient-reported outcome measures (PROMs). We aimed to assess the construct validity and internal consistency of the Quick Disabilities of the Arm, Shoulder and Hand (QuickDASH) in the pediatric population with upper extremity fractures treated both operatively and conservatively.

**Patients and methods:**

QuickDASH, along with several reference PROMs and objective outcome measures, was obtained from 148 5- to 18-year-old patients with a humeral medial epicondyle fracture or a fracture of the distal forearm in a cross-sectional setting with a single follow-up visit. Spearman’s rank correlation and linear regression models were used to assess convergent validity, exploratory factor analysis (EFA) to assess structural validity, and Cronbach’s alpha to investigate internal consistency.

**Results:**

The direction and magnitude of correlation showed by QuickDASH with reference outcome measures was consistent and demonstrated good convergent validity. EFA indicated a 3-factor model with poor fit indices and structural validity remained questionable. Construct validity was considered acceptable overall. QuickDASH demonstrated good internal consistency with an acceptable Cronbach’s alpha (α = 0.75).

**Conclusion:**

QuickDASH demonstrated acceptable construct validity and good internal consistency and is thus a valid instrument, with some limitations, to assess disability and quality of life in pediatric patients with upper extremity fractures.

Approximately 1 out of 2 children fracture at least one bone during their childhood and the majority of these fractures involve the upper extremity [1-3]. To assess optimal fracture treatment protocols, relevant and reliable outcome measures are necessary. As objective outcome measures, e.g. range of motion, can only partially describe upper extremity function, multiple patient-reported outcome measures (PROMs) have been introduced to better quantify subjective functionality [4]. There is a notable lack of validated upper extremity related PROMs, and even the most frequently used PROMs are missing high-quality validation studies [5,6]. A recent systematic review concluded that there is insufficient evidence to recommend the use of any single PROM in trials investigating childhood fractures [7].

Validation focuses on the score produced by a PROM. As the validity of a PROM is situation specific, the scores produced do not necessarily reflect the measured trait as intended when a PROM is applied to a new target population. It is therefore important to validate instruments in the specific situation in which they are intended to be used [8].

The Disabilities of the Arm, Shoulder and Hand (DASH) is a regional outcome measure of the upper extremity designed to measure physical function and symptoms [9,10]. The original 30-item DASH questionnaire was later shortened to the 11-item QuickDASH [11]. The validity, responsiveness, and reliability of QuickDASH have subsequently been demonstrated in a range of upper extremity disorders in adults [10]. Translations into several languages and cross-cultural validation studies are available [12]. There is limited evidence showing QuickDASH to be a valid outcome measure in children and adolescents with upper extremity injuries [13].

The aim of this study was to investigate the construct validity and internal consistency of QuickDASH in the pediatric population with upper extremity fractures.

## Methods

This study followed the COSMIN guidelines for studies on measurement properties of PROMs [14].

### Participants

Consecutive patients aged 5–18, with either a medial epicondyle fracture of the humerus or a distal forearm fracture involving an overriding metaphyseal radius fracture and a possible concomitant ulna fracture, injured between January 2014 and January 2021, were recruited at the New Children´s Hospital in Helsinki. The study population consisted of participants from 2 pilot studies investigating treatment results of medial epicondyle fractures [15] and distal forearm fractures [16] supplemented by patients recruited to later and still ongoing blinded randomized trials (medial epicondyle trial, trial number NCT04531085; forearm fracture trial, trial number NCT04323410). Only participants in the patient choice cohorts were included from the ongoing trials as the blinding could not be broken. Participants with QuickDASH data missing, who sustained a bilateral fracture, or were over the predefined age limit of 18 at follow-up were excluded.

Both operatively and conservatively treated patients were included. All operatively treated medial epicondyle fractures received open reduction and fixation with either cannulated screws, smooth pins, or bone anchors. The remaining patients were treated conservatively with immobilization, with either an above-elbow cast or a collar-and-cuff sling. Forearm fractures were either reduced and fixed with percutaneous smooth pins or left unreduced in an overriding position and immobilized with a cast.

Objective outcome measures and PROMs were collected at follow-up visits as dictated by the individual study protocols of the fracture trials. These visits to the outpatient clinic were conducted after a lengthy follow-up period (Table 1) as the goal was to evaluate final treatment outcomes.

**Table 1:** Demographic and clinical characteristics of participants. Values are count (%) or median (interquartile range)

Factor	Distal forearm fracture (n = 37)	Medial epicondyle fracture (n = 111)	Total (n = 148)
Sex			
Male	11 (30)	69 (62)	80 (54)
Female	26 (70)	42 (38)	68 (46)
Age at injury, years	6.8 (5.8–8.3)	12.1 (10.9–13.0)	11.4 (8.6–12.6)
Missing	0 (0)	3 (2.7)	3 (2.0)
Age at follow-up, years	9.5 (8.4–11.3)	14.0 (12.8–15.1)	13.2 (11.5–14.8)
Follow-up, years	2.6 (1.0–3.3)	1.6 (1.2–2.9)	1.9 (1.1–3.1)
Missing	0 (0)	3 (2.7)	3 (2.0)
Handedness			
Right	18 (49)	97 (87)	115 (78)
Left	2 (5)	13 (12)	15 (10)
Ambidextrous	0 (0)	1 (1)	1 (1)
Missing	17 (46)	0 (0)	17 (12)
Dominance of injured			
extremity			
Dominant	9 (24)	58 (52)	67 (45)
Non-dominant	11 (30)	53 (48)	64 (43)
Missing	17 (4)	0 (0)	17 (12)
Grip strength, kg			
Injured extremity	16.0 (12.0–18.3)	23.0 (19.0–29.0)	21.0 (16.0–28.0)
Missing	1 (2.7)	1 (0.9)	2 (1.4)
Healthy extremity	16.0 (11.0–20.0)	22.0 (18.3–29.0)	20.5 (17.0–28.0)
Missing	1 (2.7)	1 (0.9)	2 (1.4)
Difference	0 (–0.3 to 2.0)	0 (–1.8 to 2.0)	0 (–1.0 to 2.0)
Missing	1 (2.7)	1 (0.9)	2 (1.4)
Injured joint AROM,°	170 (160–185)	145 (140–152)	–
Missing	2 (5.4)	0 (0)	
Healthy joint AROM, °	174 (160–185)	150 (145–159)	–
Missing	2 (5.4)	0 (0)	
AROM difference, °	0 (–6.0 to 5.5)	–5.0 (–10.0 to 0.0)	–
Missing	2 (5.4)	0 (0)	

AROM = active range of motion. For distal forearm fractures the wrist and for medial epicondyle fractures the elbow was considered as the afflicted joint.

Difference is injured versus uninjured side.

### Patient-reported outcome measures

Participants filled out PROMs together with their guardians and received assistance in reading and understanding questionnaires if necessary.

#### QuickDASH

QuickDASH consists of 11 items and the optional sports and performing arts module (QuickDASHsp) of 4 items. Both are answered on a Likert scale of 1–5. Phrasing of the individual items is displayed in Table 2. For QuickDASHsp, no missing items were allowed. QuickDASH was considered as complete if 10 out of 11 items were answered. Final scores were calculated as suggested by the developer. The resulting scores range 0–100, with higher scores indicative of greater disability [17]. Participants could use either the Finnish [18] or Swedish [19] translation of QuickDASH. The current study employed a cross-sectional setting and replies to QuickDASH and reference outcome measures were only obtained once.

**Table 2:** Number of missing replies and number of replies with the lowest possible score (minimum) per item, mean scores, and range values (min–max), and Cronbach’s alpha for each of the items of QuickDASH and reference PROMs

Patient-reported outcome measure		Missing replies	Replies with minimum score	Score mean (range)	Cronbach’s alpha
Item	Phrasing of items				
QuickDASH (n = 148)				3.1 (0–25)	0.75
	*(No difficulty–unable)*				
Q1	Open a tight or new jar	0	130	1.13 (1–4)	0.74
Q2	Do heavy household chores (e.g., wash walls, floors)	1	139	1.06 (1–3)	0.73
Q3	Carry a shopping bag or briefcase	0	128	1.16 (1–3)	0.71
Q4	Wash your back	3	140	1.03 (1–2)	0.74
Q5	Use a knife to cut food	2	140	1.05 (1–2)	0.75
Q6	Recreational activities in which you take some force or impact through your arm, shoulder or hand (e.g., golf, hammering, tennis, etc.)	3	117	1.26 (1–3)	0.70
	*(Not at all–extremely)*				
Q7	During the past week, to what extent has your arm, shoulder, or hand problem interfered with your normal social activities with family, friends, neighbors, or groups?	0	140	1.06 (1–3)	0.75
Q8	During the past week, were you limited in your work or other regular daily activities as a result of your arm, shoulder, or hand problem?	0	140	1.06 (1–3)	0.71
	*(None–extreme)*				
Q9	Arm, shoulder, or hand pain	2	110	1.32 (1–3)	0.70
Q10	Tingling (pins and needles) in your arm, Shoulder, or hand	0	126	1.19 (1–3)	0.73
	*(No difficulty–so much difficulty that I can’t sleep)*				
Q11	During the past week, how much difficulty have you had sleeping because of the pain in your arm, shoulder, or hand?	0	143	1.03 (1–2)	0.74
QuickDASH sport & performing arts module (n = 104)				4.7 (0–100)	0.97
	*(No difficulty–unable)*				
Q1	Using your usual technique for playing your instrument or sport?	0	92	1.20 (1–5)	0.96
Q2	Playing your musical instrument or sport because of arm, shoulder, or hand pain?	0	90	1.20 (1–5)	0.97
Q3	Playing your musical instrument or sport as well as you would like?	0	91	1.20 (1–5)	0.96
Q4	Spending your usual amount of time practicing or playing your instrument or sport?	0	98	1.14 (1–5)	0.96
Pediatric Quality of Life Inventory					
Physical Health Summary Score				93 (69–100)	
Total Scale Score				91 (64–100)	
Mayo Elbow Performance Score				97 (70–100)	
Pain VAS				7 (0–92)	

VAS = visual analogue scale.

#### Mayo Elbow Performance Score (MEPS)

MEPS was available for patients with medial epicondyle fractures and was used as a reference outcome measure. MEPS focuses on elbow function and impact on activities of daily living (ADL). The produced score ranges 0–100, with lower scores indicative of greater disability [20].

#### Pediatric Quality of Life Inventory (PedsQL)

PedsQL comprises 4 sub-scales: (1) Physical Functioning, (2) Emotional Functioning, (3) Social Functioning, and (4) School Functioning. Each item is scored on a Likert scale of 0–4. The Physical Health Summary Score (PedsQL PHSS) is equivalent to the Physical Functioning sub-section score. A Total Scale Score (PedsQL TSS) can be calculated as a mean of all the items answered within subscales. Lower scores are indicative of greater disability. Both the PedsQL PHSS and the more general PedsQL TSS were chosen as reference outcome measures.

The PedsQL Pediatric Pain Questionnaire (VAS pain) assesses the amount of pain during the previous 14 days. It is scored on a visual analogue scale (VAS, 0–100), with higher scores indicative of stronger pain.

### Objective outcome measures

The active range of motion (AROM) of the affected and healthy joint, the wrist for forearm fractures and the elbow for medial epicondyle fractures, was determined using a goniometer. Grip strength was measured using a hydraulic hand dynamometer. The affected joint was compared with the healthy joint of the opposite upper extremity and the difference was recorded as the assumed change in function after injury.

### Statistics

The data are presented as means, medians with interquartile ranges (IQR), 95% confidence intervals (CI), counts with percentages, or as ranges. The statistical analysis was performed using R version 4.3.0 (R Foundation for Statistical Computing, Vienna, Austria).

A response to a single item of the QuickDASH questionnaire was missing for 11 participants (11 of 1,628 item responses, 0.67%) (Table 2). A mean imputation method was used for the missing item scores, ensuring that the calculated total QuickDASH scores remained unaffected. A sample size of over 100 participants was considered adequate for all analyses performed as outlined by the COSMIN Study Design checklist [21].

We hypothesized that the correlation of QuickDASH with the reference PROMs MEPS and PedsQL PHSS would be large, as they have been designed to measure a similar construct. As the PedsQL TSS measures quality of life with a more general scope, we hypothesized that it would correlate moderately with QuickDASH. As QuickDASH scores increase and PedsQL and MEPS scores decrease with worse function, the correlations were expected to be negative. We hypothesized that pain would have a considerable impact on upper extremity function, and therefore VAS pain was hypothesized to have a moderate to large positive correlation with QuickDASH. Finally, reductions in comparison with the healthy extremity in the objective outcome measures—joint AROM and grip strength—were hypothesized to have a moderate negative correlation with QuickDASH.

Convergent validity of QuickDASH was assessed by investigating the relationship of QuickDASH with several reference outcome measures using correlation coefficients. Scatterplots showed monotonic relationships. Spearman’s rank correlation coefficient was used to calculate the non-adjusted correlations. Values of P ≥ 0.7 or P ≤ –0.7 were hypothesized to represent large, 0.7 > P ≥ 0.5 or –0.7 < P ≤ –0.5 moderate, and 0.5 > P ≥ 0.3, or –0.5 < P ≤ –0.3 small correlation.

Linear regression models were applied to measure correlation with adjustment for sex. Linear regression models were applied to the whole study sample as well as group-wise to adolescents (aged 13–18, n = 79) and younger children (aged 5–12, n = 69). Correlations were assessed using regression β coefficients. Units of standard deviation were used to measure β. Values of β ≥ 0.50 or β ≤ –0.50 represent large, 0.3 ≤ β < 0.5 or –0.5 > β ≥ –0.3 moderate, and 0.1 ≤ β < 0.3 or –0.3 > β ≥ –0.1 small correlation. Convergent validity of QuickDASHsp was assessed in a similar fashion. Missing reference outcome measure data were handled with listwise deletion. Durbin–Watson tests did not show autocorrelation in any of the models. Residuals vs Fitted, Scale-Location, and Q–Q Residuals plots were investigated and a Breusch–Pagan test was employed for all models. The linearity assumption was met in most models. Issues with heteroscedasticity were observed in multiple models and this violation of the homoscedasticity assumption had to be accepted.

Structural validity was assessed using exploratory factor analysis (EFA). The suitability of the sample for EFA was assessed using the Kaiser–Meyer–Olkin test (KMO). The number of factors was estimated by examining scree plots, using Cattell’s test and parallel analysis. Multivariate normality was assessed using Mardia’s test and a chi-square Q–Q plot, both of which showed non-normality. The Anderson–Darling test and distribution histograms for individual item responses also showed univariate non-normality. A Pearson correlation matrix was used as the matrix of association. Principal axis factoring was chosen as the extraction method, as this makes no assumption on data distribution and is suitable for use with ordinal data. A high correlation among factors in the multifactorial model was expected, and an oblique Promax rotation was applied. Model fit was assessed using the RMSEA index and the Tucker–Lewis index (TLI). Threshold values for acceptable fit were determined as RMSEA index < 0.08 and TLI > 0.9.

Internal consistency of QuickDASH was assessed by calculating the Cronbach’s alpha coefficient. A threshold value of 0.70 was considered acceptable. Floor and ceiling values were calculated by dividing the number of obtained maximum or minimum scores by the total number of questionnaires completed.

### Ethics, funding, and disclosures

The study was approved by the HUS Regional Committee on Medical Research Ethics (HUS/1443/2019, HUS/2345/2019). Written informed consent was obtained from all participants. This research received no specific grant from any funding agency. The authors have no conflicts of interest to declare. Complete disclosure of interest forms according to ICMJE are available on the article page, doi: 10.2340/17453674.2024.40181

## Results

Of the recruited 182 participants, 140 had a medial epicondyle fracture of the humerus and 42 a distal forearm fracture. QuickDASH data was missing for 27 participants, 1 participant had a bilateral epicondyle fracture, and 6 participants were over the predefined age limit. The final sample consisted of 148 patients.

Basic demographics and clinical features along with the measured objective outcome measures at follow-up showed the age at PROM administration was 13 (SD 2.6, range 5.3–17). Participants with medial epicondyle fractures accounted for 75% (n = 111) and participants with forearm fractures for 25% (n = 37) of the study population (Table 1). Medial epicondyle fractures were treated operatively in 52% of cases (n = 55) and forearm fractures in 47% of cases (n = 18) with the remaining participants receiving conservative treatment. The mean score of QuickDASH was 3.1 and ranged only 0–25. None of the participants reported 5, indicative of the highest disability, on the Likert scale on any of the individual items. QuickDASHsp mean score was low at 4.7. QuickDASHsp, however, ranged the full 0–100. Reference PROMs similarly showed average scores indicative of low dysfunction (Table 2).

There was a high number of minimum scores for all items of QuickDASH and QuickDASHsp (Table 2). For QuickDASH, the ceiling effect was calculated at 59% (88/148). No maximum scores and no floor effect for QuickDASH were observed. The QuickDASHsp module had a ceiling effect of 85% (88/103) and a floor effect of 2% (2/103) (Table 2).

In the unadjusted analyses using Spearman’s rank correlation coefficients (Table 3), a statistically significant correlation between QuickDASH and the reference PROMs PedsQL PHSS, PedsQL TSS, VAS pain, and MEPS was observed. Although the change in elbow AROM showed a statistically significant correlation with QuickDASH, a change in wrist AROM or grip strength did not. QuickDASHsp showed similar correlations with reference outcomes except for the PedsQL TSS, which showed no correlation. After adjusting for sex and subgrouping into adolescents and younger children, correlations remained largely unaffected in the linear regression models (Figure).

**Table 3:** Spearman’s rank correlation coefficients (ρ)

	QuickDASH		QuickDASH sport & performing arts module	
	ρ	P value	ρ	P value
Change compared with healthy side				
AROM injured elbow	–0.37	< 0.001	–0.39	< 0.001
AROM injured wrist	–0.15	0.4	–0.05	0.8
Grip force injured extremity	–0.10	0.3	–0.10	0.3
Pediatric Quality of Life Inventory				
Physical Health Summary Score	–0.62	< 0.001	–0.51	< 0.001
Total Scale Score	–0.40	< 0.001	–0.12	0.3
VAS pain	0.66	< 0.001	0.50	< 0.001
Mayo Elbow Performance Score	–0.40	< 0.001	–0.53	< 0.001

AROM = active range of motion, VAS = visual analogue scale.

**Figure:**
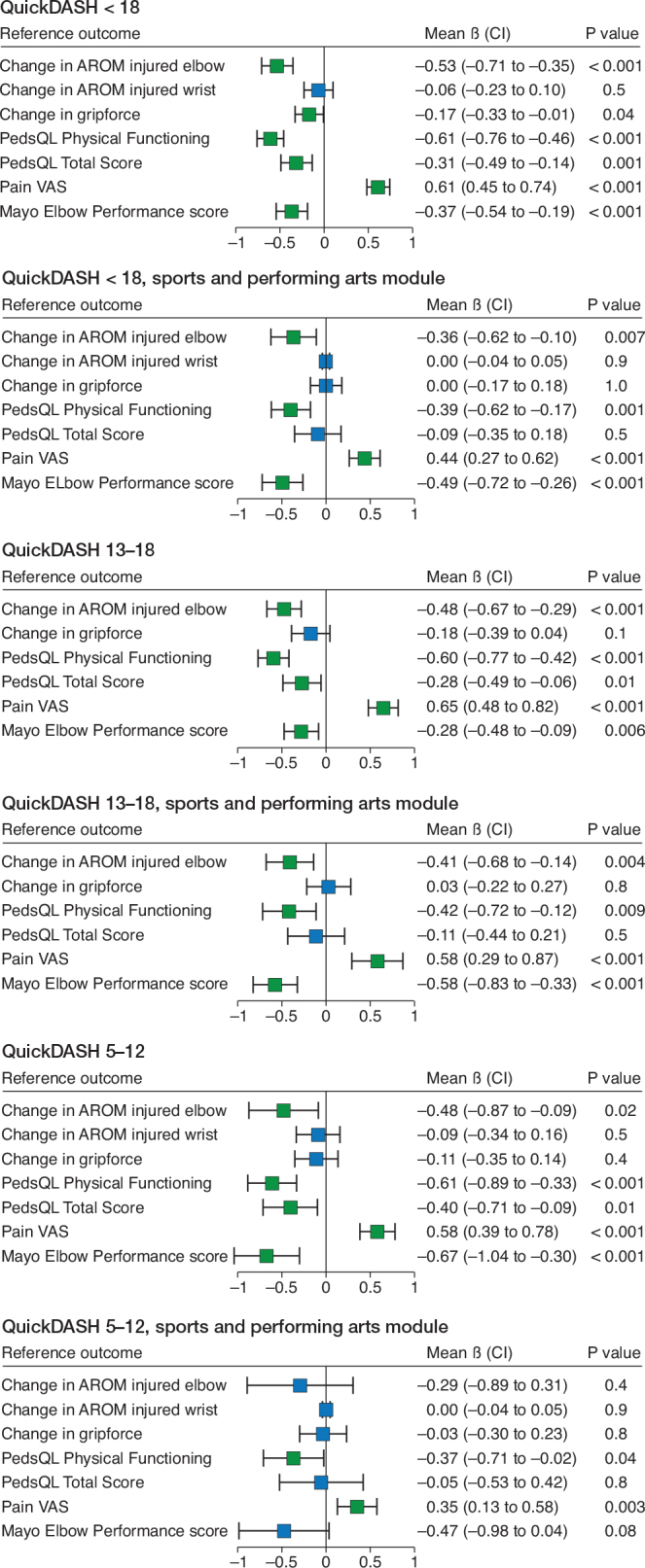
Correlation of QuickDASH with reference outcome measures. Sex-standardized correlation regression ß is displayed on the x-axes. Boxes are mean values and whiskers 95% confidence intervals (CI). Change of AROM = change in active range of motion compared with unaffected side. PedsQL = Pediatric Quality of Life inventory. Pain VAS = Pediatric Questionnaire visual analog scale. .

The KMO test resulted in a value of 0.69 for QuickDASH, indicating acceptable sampling. Cattell’s scree plot test and parallel analysis pointed to a 3-factor model. A 1-factor EFA was first conducted. All items showed meaningful factor loading except items 5 and 7. Model fit was poor, RMSEA index = 0.20 and TLI = 0.36, and only 26% of variance was explained by this factor.

In EFA with a 3-factor model, the first factor accounted for 21%, the second 15%, and the third 13% of variance, resulting in a cumulative variance of 49%. Items 1–5 loaded mainly on the first factor. Item 3, however, showed splitting among the first and second factor, and the factor loading for item 5 was weak. Items 6, 9, and 10 loaded on the second factor. Finally, items 7, 8, and 11 loaded on the third factor. Although model fit was greatly improved in comparison with the 1-factor model, it remained unacceptable with TLI = 0.80 and RMSEA index = 0.11, CI 0.08–0.14 (Table 4).

**Table 4:** Factor loadings of the exploratory factor analysis. Factor loading cut off = 0.3

Item	3-factor model			1-factor model
	Factor 1	Factor 2	Factor 3	Factor 1
QuickDASH				
Q1	0.35	0.09	0.14	0.47
Q2	1.05	–0.30	0.16	0.64
Q3	0.48	0.34	–0.10	0.64
Q4	0.90	–0.14	–0.01	0.59
Q5	0.23	0.19	–0.15	0.28
Q6	0.01	0.75	0.02	0.61
Q7	–0.20	0.10	0.63	0.23
Q8	0.12	0.12	0.78	0.55
Q9	–0.00	0.69	0.17	0.64
Q10	–0.16	0.64	0.07	0.41
Q11	0.16	–0.02	0.42	0.34
QuickDASH sport & performing arts module				
Q1				0.95
Q2				0.93
Q3				0.96
Q4				0.94

For QuickDASHsp, the KMO test indicated good sampling at 0.86. Cattell’s scree plot test and parallel analysis pointed to a 1-factor model. In EFA, all items loaded heavily on 1 factor. The factor accounted for 89% of variance. TLI = 0.95 indicated good model fit. In contrast, RMSEA index = 0.20 (CI 0.10–0.33) indicated poor fit.

The internal consistency of QuickDASH was considered acceptable with Cronbach’s alpha at 0.75. When items were excluded one by one, Cronbach’s alpha ranged from 0.70 to 0.75. The QuickDASHsp module showed high internal consistency with alpha = 0.97. When items were excluded one by one, alpha ranged from 0.96 to 0.97 (see Table 2).

## Discussion

In our study, 2 aspects of construct validity, convergent and structural validity, were assessed when QuickDASH was applied to a pediatric population with upper-extremity fractures. The magnitude and direction of correlation of QuickDASH with reference outcome measures showed acceptable convergent validity. However, structural validity was more questionable, as QuickDASH seemed to have a multidimensional as opposed to a unidimensional structure as would be expected for a PROM designed to measure a specific single trait. QuickDASH showed acceptable levels of internal consistency.

Convergent validity was assessed by exploring correlations of QuickDASH with reference outcome measures. The validity of PedsQL and VAS pain in pediatric populations in general [22,23] and in an orthopedic setting [24] have been demonstrated previously: the PedsQL PHSS as well as VAS pain showed a strong correlation with QuickDASH. In contrast, the more general PedsQL TSS showed a moderate correlation with QuickDASH. The magnitude and direction of these correlations were as hypothesized a priori. Previous evidence seems to be in line with our findings, as a similar correlation between PedsQL and QuickDASH was observed in a pediatric population with upper extremity injuries [13].

There are no validation studies available for MEPS in pediatric populations. This lack of evidence diminishes the value of MEPS as a reference outcome measure. It was still used as there are currently no validated elbow-specific PROMs available for children. Even though there was a statistically significant correlation between QuickDASH and MEPS, it remained only moderate and was weaker than hypothesized a priori. To some extent, this might reflect the poor performance of the reference outcome measure MEPS in assessing dysfunction in our sample.

Objective outcome measures did not correlate with QuickDASH as strongly as predicted. Indeed, only the change of elbow AROM showed a strong correlation with QuickDASH. Contrary to our a priori hypothesis, the change in wrist AROM showed no correlation and grip strength only a weak correlation with QuickDASH. The follow-up times in our sample were long and even the largest reductions in AROM relatively small. A possible explanation for the lack of correlation might be that pediatric patients simply healed remarkably well or were able to adapt to the loss in function. It is also of note that all participants with forearm fractures were in the group of younger children (5–12 years old). Therefore, the observed lack of correlation with wrist AROM could also indicate the poorer performance of QuickDASH in younger children.

When the sample was grouped age-wise into adolescents and younger children, the trends of correlation remained the same. PedsQL PHSS and VAS pain showed a strong correlation and PedsQL TSS and elbow AROM a moderate correlation with QuickDASH in both age groups. MEPS correlated moderately with QuickDASH in adolescents and strongly in younger children. The confidence intervals were somewhat wider in the group of younger children, possibly indicating greater fluctuation in QuickDASH scores.

QuickDASHsp seemed to show similar correlations with reference PROMs to the main QuickDASH questionnaire, although the strength of correlation was weaker throughout and confidence intervals were wider. This might be due to the limited number of completed questionnaires. The most notable difference was a lack of correlation between QuickDASHsp and the PedsQL TSS, which is likely explained by the difference in the construct measured. PedsQL TSS encompasses a wider array of ADLs as opposed to the focus on high-demand activities measured in QuickDASHsp. A modest decline in function could have a significant impact on high-demand activities without affecting common ADLs.

For QuickDASH, a unidimensional structure is expected as a single total score is produced and there are no defined subscales. The individual items of QuickDASH encompass differing aspects of function and disability and therefore exhibit a multidimensional structure. The structural validity and dimensionality of QuickDASH has not been investigated in pediatric settings and the unidimensional structure has been questioned in adult populations [25-27]. In our sample, the 1-factor model in EFA showed meaningful factor loadings on all items except 5 and 7. The fit indices, however, showed poor model fit and the variance explained by the factor remained unacceptably low. Parallel analysis and scree plots suggested a 3-factor model. In this model, the first factor loaded on items 1 to 5, the second on items 6, 9, and 10, and the third on items 7, 8, and 11. We hypothesize that the construct represented by factor 1 is physical ability. Items that loaded on factor 2 describe pain, sensory changes, and inability to withstand force and the represented construct seems to be symptoms. The third factor represents negative impact on ADLs. Splitting among factors was observed for items 3 and 5. Factor loadings were relatively weak for some items. This was especially evident for items 1 and 5. Assessing the content validity of QuickDASH was beyond the scope of our study. However, this heavy splitting among factors and weak factor loadings for some items could indicate that these items are irrelevant when QuickDASH is applied to pediatric populations with upper extremity fractures.

QuickDASHsp seemed to have a unidimensional structure in EFA. All items loaded heavily on a single factor and a significant portion of variance was explained by this factor. TLI showed good model fit as opposed to the RMSEA index, which did not.

### Limitations

The long follow up-time is a significant limitation of our study. The evidence provided by our study supports the validity of QuickDASH only when assessing final outcomes. Furthermore, QuickDASH and the QuickDASHsp module both showed a significant ceiling effect in our sample with a high number of low scores showing no dysfunction. This might be indicative of the PROMs’ poor ability to identify upper extremity dysfunction in our setting. As pediatric populations show exceptional healing after fractures [28] and all reference outcome measures also indicated low disability, it is likely that the observed ceiling effect was also caused by the long follow-up time. Whether the significant ceiling effect in our sample is truly due to full recoveries made by the participants, or if it reflects poor performance of QuickDASH in this long-term follow-up setting, remains unclear.

The youngest participants were not able to read the questionnaires on their own and received help from guardians, which might introduce proxy response bias [29]. Whether the participants had received help responding to the questionnaires was not recorded, which precluded the possibility of subgroup analysis with exclusion of these participants. The youngest participants were over 5 years old at follow-up. Our results are therefore not generalizable to the youngest children. Moreover, the wording and concepts of some items of QuickDASH might be irrelevant or too complicated to understand for the youngest age groups included in the study sample. Further study on the content validity in the youngest age group is warranted. Although QuickDASH has been designed to assess outcomes in patients with shoulder pathologies, these were not included in our study, and no evidence on validity in this patient group is provided.

A further limitation of our study is the lack of repeat testing of participants due to the cross-sectional study design. The test–retest reliability or responsiveness could therefore not be evaluated.

### Conclusions

QuickDASH demonstrated acceptable construct validity and good internal consistency and can be considered a valid instrument, with some limitations, to assess disability and quality of life in pediatric patients with upper extremity fractures.

## Notes

NK, SH, PG, TL, and JR designed the study protocol. PG and TL participated in data acquisition. Data analysis and manuscript writing was undertaken by NK. Data interpretation was done by NK, SH, and JR. All authors participated in manuscript editing.

Handling co-editors: Jeppe Vejlgaard Rasmussen and Robin Christensen

*Acta* thanks Kristofer Hallberg and Jesper Ougaard Schønnemann for help with peer review of this manuscript.
